# Comparison of Pudendal Nerve Block and Spinal Anesthesia in Proctological Surgeries: Efficacy, Safety, and Patient Outcomes

**DOI:** 10.3390/medicina60101651

**Published:** 2024-10-09

**Authors:** Samet Şahin, Cenk Yazkan, Özcan Dere, Ilgaz Kayılıoğlu, Önder Özcan

**Affiliations:** Department of General Surgery, School of Medicine, Muğla Sıtkı Koçman University, Muğla 48121, Türkiye; cenkyazkan@mu.edu.tr (C.Y.); ozcandere@mu.edu.tr (Ö.D.); kayilioglu@gmail.com (I.K.); onderozcan@mu.edu.tr (Ö.Ö.)

**Keywords:** proctological surgery, pudendal nerve block, efficacy

## Abstract

*Background and Objectives:* The selection of an appropriate anesthesia method is a critical factor in the surgical treatment of proctological diseases, significantly impacting patient outcomes and comfort. Pudendal nerve block (PNB) and spinal anesthesia (SA) are commonly employed in these surgeries, yet the optimal choice between the two remains debated. This study aims to compare the efficacy and safety of PNB and SA in patients undergoing surgical treatment for various proctological conditions, with a focus on postoperative pain management, functional outcomes, and complication rates. *Materials and Methods:* A prospective observational study was conducted on 590 patients who underwent proctological surgery under either PNB (n = 435) or SA (n = 155). Pain levels were assessed using the Visual Analog Scale (VAS), while functional outcomes were measured using the World Health Organization Disability Assessment Schedule (WHODAS 2.0). Statistical analysis was performed to compare the outcomes between the two groups. *Results:* Patients in the PNB group reported significantly lower postoperative VAS scores compared to those in the SA group, particularly in hemorrhoidectomy and laser hemorrhoidoplasty procedures. The PNB group also demonstrated superior functional outcomes, with lower postoperative WHODAS 2.0 scores and a reduced incidence of urinary retention compared to the SA group. Furthermore, the duration of surgery and hospital stay were significantly shorter for patients in the PNB group. *Conclusions:* The findings suggest that PNB may offer advantages over SA in proctological surgeries, particularly in terms of pain management, functional recovery, and reduced complication rates. PNB should be considered a viable alternative to SA, particularly in cases where rapid recovery and minimizing complications are priorities. Exceptions to this include specific proctological surgeries, such as those for malignant tumors in the region, complex anal fistulas, proctological conditions arising from inflammatory bowel diseases, and patients on immunosuppressive therapy. Further research is needed to confirm these results and optimize anesthesia selection in this context.

## 1. Introduction

The treatment of proctological diseases, which include conditions such as hemorrhoids, anal fissures, anal fistulas, and rectal prolapse, poses significant challenges in the field of colorectal surgery, affecting millions of individuals globally and considerably impacting their quality of life [1]. Hemorrhoids, for example, are estimated to affect approximately 4.4% of the global population, with a higher prevalence in individuals over the age of 45. Anal fissures, although less common, have an incidence rate of around 1 per 350 people annually. Anal fistulas affect about 1.04 to 2.32 per 10,000 individuals, often requiring surgical intervention. Rectal prolapse, though rarer, predominantly occurs in elderly patients, with an estimated incidence of 2.5 cases per 100,000 people. These conditions frequently necessitate surgical treatment, and the choice of anesthesia plays a pivotal role in determining both the surgical outcome and patient comfort, particularly in facilitating a quicker recovery [1,2,3].

Recent studies have explored various anesthesia techniques to enhance patient outcomes and reduce postoperative pain in proctological surgeries. For instance, tailored anal block (TAB) has been introduced as a novel anesthesia method, showing effectiveness in managing hemorrhoid cases in outpatient settings, potentially reducing the hospital stay and accelerating recovery [4]. Additionally, the application of local anesthesia in proctological surgeries has been reported to be effective in terms of patient comfort and surgical success [5].

Pain and disability are subjective concepts that directly influence patients’ postoperative experiences. Therefore, objective assessment of these experiences is crucial for accurately measuring surgical outcomes. The Visual Analog Scale (VAS) is frequently used to quantitatively assess pain levels. VAS allows patients to rate their pain on a specific scale, providing a more concrete representation of their pain experiences [6]. Similarly, the World Health Organization Disability Assessment Schedule (WHODAS 2.0) offers a standardized method for measuring disability in daily life activities. WHODAS 2.0 is a comprehensive tool for evaluating individuals’ physical and mental functions and helps objectively assess the impact of surgical interventions on patients’ overall quality of life [7].

Among the anesthesia methods used in proctological surgeries, pudendal nerve block (PNB) and spinal anesthesia (SA) are widely preferred. PNB provides more localized anesthesia, offering more targeted pain control during surgery, and is particularly used to reduce the need for postoperative analgesics [2,8]. Moreover, studies indicate that preemptive analgesia in anorectal surgeries can significantly decrease postoperative pain, highlighting the importance of the anesthesia choice in improving patient outcomes [9]. SA, on the other hand, is effective over a broader area, providing complete sensory loss in the lower body. Both methods have their advantages over each other, but the question of which method is more suitable for specific surgical conditions remains a subject of debate in the literature [10].

In the literature, the use of local anesthesia in office procedures for the surgical treatment of proctological diseases is common. However, in these types of procedures, the anesthesia applied is often limited to the area to be operated on, and there are limited data on office procedures performed solely with PNB without systemic anesthetics. Only Bonatti et al. presented data on 36 proctological operations performed with PNB [3]. Additionally, a single-center study emphasized that various techniques of local anesthesia could effectively manage proctological conditions in an outpatient setting, suggesting local anesthesia as a feasible option for such interventions [9].

The aim of this study is to compare the efficacy and safety of pudendal block and spinal anesthesia in patients undergoing surgical treatment for various proctological diagnoses. In our study, we aim to provide scientific evidence for the optimal anesthesia selection in proctological surgeries by comparing the effects of these two anesthesia methods on surgical success, postoperative pain management, and patient satisfaction.

## 2. Materials and Methods

### 2.1. Study Design

This study was a prospective, observational study. The study was developed and presented according to the Strengthening the Reporting of Observational Studies in Epidemiology (STROBE, Supplementary file S1) guidelines. The study was conducted in accordance with the principles of the Declaration of Helsinki and Good Epidemiological Practices (World Medical Association. World Medical Association Declaration of Helsinki: ethical principles for medical research involving human subjects) [11].

The study was approved by an independent ethical committee (Izmir Bakircay University Ethics Committee, Decision No: 1407, Research No: 1387), and written informed consent was obtained from all patients. All operations were performed by the same surgeon, who followed standard surgical procedures. When the decision to operate on the patients was made, all patients were placed on the same appointment list. They were invited to the hospital for the operation from this list, and when the surgeon was in the proctology unit, the operations were performed under PNB, and when he was in the operating room, the operations were performed under SA. No patient-identifying data (name, date of birth, address, telephone number, etc.) were recorded.

### 2.2. Patient Selection Criteria

Inclusion criteria: Patients aged 18 to 100 years who were diagnosed with benign proctological diseases and scheduled for surgery and who consented to participate in the study were included.

Exclusion criteria: Patients under the age of 18 and patients in whom sufficient pain palliation was not achieved after PNB and who subsequently had their procedure terminated (surgery was not performed because pain palliation could not be achieved) were excluded from the study. We excluded cases where the pudendal nerve block was inadequate due to factors such as anatomical variations or technical difficulties to ensure consistent anesthesia outcomes and avoid confounding factors that could affect the validity and comparability of the results. Also, patients with preoperative pelvic floor dysfunction (e.g., rectocele, fecal incontinence, obstructive defecation syndrome, internal/external rectal prolapse, pelvic descent, chronic constipation, etc.), patients with anal stenosis due to previous proctological surgery, paraplegic patients, patients diagnosed with inflammatory bowel disease (IBD), patients with a malignancy of the anal region, and patients whose data could not be obtained were excluded from the study.

### 2.3. Variables and Definitions

Proctological diseases such as hemorrhoidal disease, anal fistula, anal fissure, anal wart, and anal abscess surgeries were evaluated.

**Figure 1:**
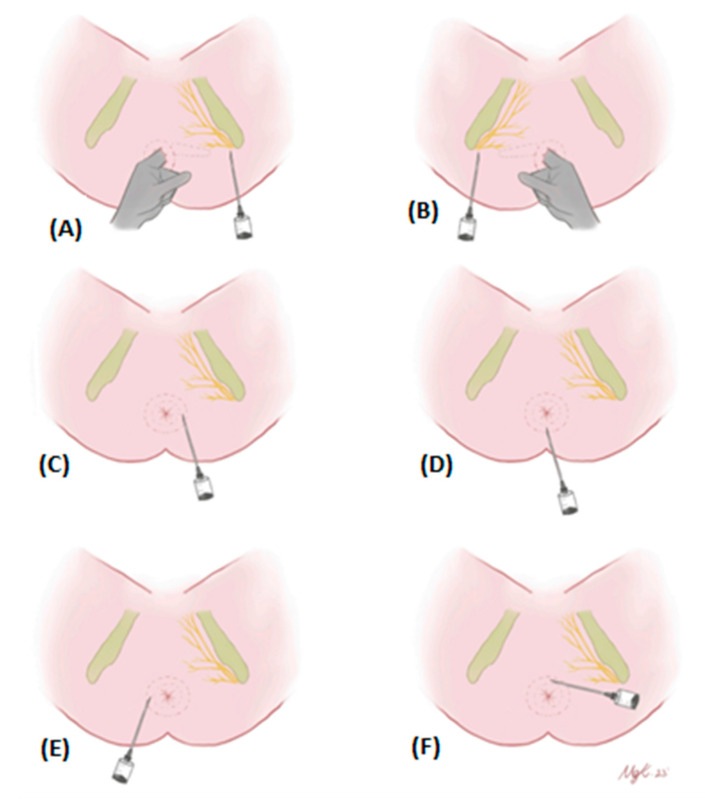
PNB application. Lidocaine diluted 1:1 with 0.9% saline was used for PNB. Initially, 4 mL of the mixture was injected into each pudendal nerve trace by palpating both ischial tuberosities outward at a 45-degree angle (**A**,**B**). Then, the intersphincteric sulcus was observed with digital rectal examination, and the anesthesia was terminated by injecting 2 mL of local anesthetic into each quadrant (3–6–9–12 o’clock positions) (**C**–**F**).

For the pudendal nerve block (PNB), a solution of lidocaine diluted 1:1 with 0.9% saline was prepared. Initially, the patient was positioned appropriately to allow for access to the pudendal nerve (lithotomy position). Using anatomical landmarks, both ischial tuberosities were palpated, and the injection sites were identified by locating the pudendal nerve traces at a 45-degree angle outward from the tuberosities. A total of 4 mL of the anesthetic solution was injected into each pudendal nerve trace bilaterally. After this, a digital rectal examination was performed to palpate the intersphincteric sulcus and internal sphincter. The anesthesia was then completed by injecting 2 mL of the local anesthetic into each of the four quadrants of the anal canal (at the 3, 6, 9, and 12 o’clock positions). This multi-step approach ensured comprehensive anesthesia coverage for the procedure (Figure 1).

The following variables were assessed: age, gender, disease diagnosis, type of anesthesia, type of surgery performed, duration of surgery, postoperative hospital follow-up duration, long-term follow-up duration, and any recurrence or complications that occurred during this period. The preoperative, postoperative 1st week, and postoperative 1st month VAS scores, as well as the preoperative and postoperative 1st month WHODAS 2.0 scores, were recorded for all patients.

The preoperative, intraoperative, and postoperative management and surgical approaches for the patients are summarized in Table 1.

### 2.4. Study Setting

This study was conducted in the only tertiary hospital in Muğla. Since it is the reference hospital of the region, an average of 30 proctology patients apply to the outpatient clinic per day, and approximately 20 proctology surgeries are performed per week.

### 2.5. Statistical Analysis

The sample size was calculated using G * Power 3 software (Institute of Experimental Psychology, Heinrich Heine University, Düsseldorf, Germany). A power analysis calculated the minimum sample size required to detect a statistical difference at a medium effect size (Cohen’s d: 0.5) and a 95% power and α = 0.05 significance level as 296 participants, with a minimum of 148 in each group. There was a numerical difference between the PNB and SA groups, because the patient operations continued in the PNB group until a sufficient sample was created in the SA group.

Statistical analyses were performed using SPSS software (version 24.0, IBM Corp., Armonk, NY, USA). The normality of the data distribution was evaluated using the Kolmogorov–Smirnov and Shapiro–Wilk tests. Descriptive statistics were expressed as mean ± standard deviation for normally distributed data and as median (interquartile range) for non-normally distributed data. The chi-square or Fisher’s exact tests were used for the comparison of categorical data, while the independent samples *t*-test or Mann–Whitney U test was applied for the comparison of continuous variables between independent groups. The Friedman test was used for the comparison of pre- and post-scale scores in dependent groups, and the paired *t*-test or Wilcoxon signed-rank test was used for preoperative–postoperative comparisons. A *p*-value of less than 0.05 was considered statistically significant.

## 3. Results

**Figure 2:**
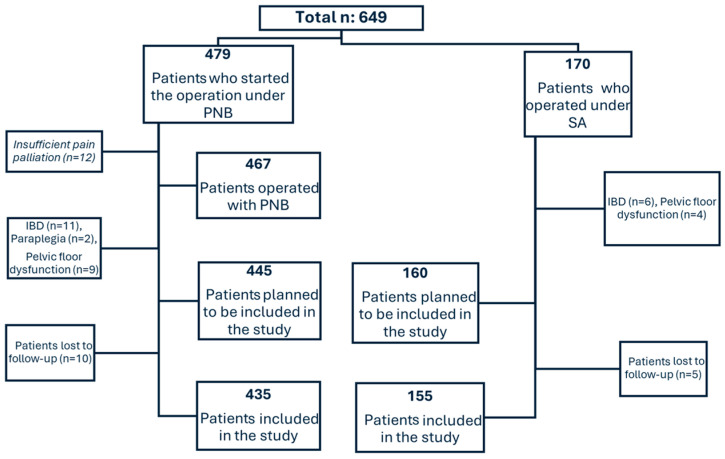
Determination of the sample according to the inclusion and exclusion criteria.

A total of 590 patients, out of 649 initially considered, were included in this study—435 under the PNB group and 155 under the SA group following the exclusion criteria (Figure 2).

### 3.1. Baseline Characteristics

The mean age was 44.42 ± 13.62 years, with 25.4% female and 74.6% male patients. The gender and age distributions were comparable between the groups (*p* = 0.592 and *p* = 0.189). The median follow-up period was 16 months, with no significant difference between groups (*p* = 0.670). Descriptive analyses by anesthesia type are provided in Table 2.

### 3.2. Postoperative Pain Outcomes

The preoperative Visual Analog Scale (VAS) scores were similar across both groups (*p* > 0.05 for all parameters, Table 3). The postoperative 1st-week VAS scores showed no significant increase compared to the preoperative scores in either group (*p* > 0.05). By the 1st month, the VAS scores significantly decreased from the preoperative and 1st-week levels in both groups (*p* < 0.001). Comparatively, the postoperative VAS scores for hemorrhoidectomy and laser hemorrhoidoplasty (LHP) were lower in the PNB group after both the 1st week and 1st month (Table 3).

For anal fistula surgeries, the postoperative 1st-week VAS scores were higher than the preoperative scores in both groups, while the 1st-month scores were significantly lower (*p* < 0.05). Drainage seton placement resulted in significantly lower 1st-month VAS scores compared to the preoperative scores in both groups (*p* < 0.001). PNB patients experienced less pain in the 1st week compared to SA patients (*p* < 0.001). Additional VAS comparisons for other conditions are presented in Table 3.

### 3.3. Postoperative Functional Outcomes

The preoperative WHODAS 2.0 scores showed no significant difference between groups (*p* > 0.05, Table 4). The postoperative WHODAS scores were significantly lower than the preoperative scores in patients undergoing hemorrhoidectomy, LHP, hemorrhoidopexy, and thrombectomy under PNB (*p* < 0.001, *p* < 0.001, *p* = 0.043, *p* = 0.004). In the SA group, only hemorrhoidectomy showed a significant increase in postoperative WHODAS scores (*p* = 0.009). PNB patients had significantly lower postoperative WHODAS scores compared to SA patients (*p* = 0.009, Table 4).

For anal fistula surgeries, the postoperative WHODAS scores were significantly lower than the preoperative scores for both the PNB and SA groups, except in the SA group for LAFT (*p* > 0.999). Comparatively, PNB patients showed significantly lower postoperative WHODAS scores for fistulotomy and seton placement than SA patients (*p* = 0.047, *p* = 0.029, Table 4).

### 3.4. Early Complications, Surgery Duration, and Hospital Stay

Urinary retention was significantly higher in the SA group across all diagnoses and surgeries compared to PNB (*p* < 0.001, Table 5). Surgery duration and hospital stay were shorter in the PNB group (*p* < 0.001, Table 6).

## 4. Discussion

The optimal anesthesia technique for proctologic surgeries remains controversial. Our study compared the efficacy and safety of PNB and SA in proctologic surgeries. The findings suggest that PNB may provide more favorable results than SA, especially in terms of postoperative pain management, functional outcomes, and complication rates.

### 4.1. Pain Management

Postoperative pain management is an important issue for both the patient and the physician after proctology surgeries. In our study, we found that the patients in the PNB group had significantly lower Visual Analog Scale (VAS) scores compared to those in the SA group. This difference was particularly evident in hemorrhoidectomy and LHP surgeries. In the follow-up of these surgeries, we observed that the PNB group exhibited better pain control both in the first postoperative week and in the first postoperative month. Similar results were reported in previous studies, arguing that PNB directly targeting the pudendal nerve resulted in better analgesia compared to SA [12,13,14].

This superior pain relief observed with PNB might be attributed to its ability to block nociceptive signals, specifically from the anal and perineal regions, without affecting the entire lower body as SA does. The reduction in postoperative pain could potentially reduce the need for additional analgesics, thus minimizing the risk of opioid-related side effects [15].

### 4.2. Functional Outcomes

An important component of postoperative recovery in proctology is functional recovery. In our study, we found that the postoperative WHODAS 2.0 scores were significantly lower in the PNB group. This suggests that PNB may facilitate a more rapid return to baseline functional status, which aligns with the existing literature. For instance, a systematic review by Sammour et al. indicated that using PNB instead of spinal anesthesia for hemorrhoidectomy was associated with improved pain management and functional outcomes [16].

Moreover, Imbelloni et al. conducted a controlled clinical study demonstrating that bilateral PNB resulted in significantly prolonged perineal anesthesia compared to those receiving other forms of anesthesia [17]. Similarly, Aldabbas et al. found that combining PNB with general anesthesia led to extended pain-free durations [18].

Furthermore, the study by Alkhaldi et al. highlights that PNB can provide significant improvements in postoperative outcomes, including pain management and return to normal activities [19]. These findings are particularly consistent with our study, as they underscore the potential of PNB to enhance recovery in patients undergoing anorectal surgery.

### 4.3. Complications

In our study, the incidence of urinary retention was higher in the SA group. This finding, especially observed after fistulotomy and seton placement surgeries, aligns with the current literature. It is known that SA can negatively affect bladder function. [20]. In contrast, the localized nature of the PNB appears to reduce this risk, making it a safer alternative regarding urinary complications. Studies have indicated that PNB is associated with a lower incidence of urinary retention compared to spinal anesthesia, as it does not produce the same degree of neural blockade affecting bladder control [19,21,22].

In our study, we noted that both anesthesia methods, PNB and SA, were generally safe for patients undergoing proctologic surgery. The absence of significant differences in infection rates or wound healing complications suggests that PNB is as safe as SA. Similarly, Slopnik et al. demonstrated in a randomized controlled trial that PNB provided similar results in terms of infection and wound healing compared to patients receiving general anesthesia for vaginal surgeries [23]. Additionally, Yasrab et al. found that bilateral PNB application after endoscopic bladder interventions significantly reduced catheter-related bladder discomfort without any adverse effects [24]. In two studies evaluating postoperative complications in anorectal surgeries, it was reported that while reasonable treatment responses were obtained in patients who underwent PNB, the complication rates did not change [18] or decrease [16]. This supports the idea that PNB is a viable and safe alternative, especially for patients who are at higher risk of complications from more invasive anesthesia techniques.

### 4.4. Limitations

Despite the strengths of this study, several limitations must be acknowledged. The unequal distribution of patients across various surgical procedures may affect the statistical power of our comparisons, potentially limiting the generalizability of our findings. Additionally, the relatively small sample sizes for certain procedures, such as hemorrhoidopexy and thrombectomy, prevent definitive conclusions for these specific surgeries.

Another limitation is the absence of a cost–benefit analysis, which could provide valuable insights into the economic advantages of PNB over SA, particularly given the shorter hospital stays and potentially reduced postoperative care associated with PNB. Furthermore, the lack of preoperative WHODAS scores for patients undergoing emergency procedures like abscess drainage represents a missed opportunity to more comprehensively assess functional recovery.

It should also be noted that PNB may not provide adequate anesthesia in some patients, which might have influenced the decision to avoid its use in certain cases. Although the same surgical procedures were compared, variations in disease severity could have affected the choice of anesthesia. Therefore, it is crucial that these findings be re-evaluated through randomized controlled trials.

## 5. Conclusions

In conclusion, this study provides evidence that PNB may offer several advantages over SA in proctological surgeries, particularly in terms of pain management, functional recovery, and the incidence of urinary retention. These findings suggest that PNB should be considered a viable alternative to SA, especially in cases where rapid recovery and minimizing complications are prioritized. Further research with larger, more diverse patient populations and detailed cost analyses is necessary to confirm these results and refine the guidelines for anesthesia selection in proctological surgeries.

We must clearly state that the message of the study is by no means to represent pudendal block as a superior procedure to spinal anesthesia. It simply suggests that many proctologic operations can be safely performed with only pudendal block in appropriate patients. We believe that with sufficient experience, this anesthesia procedure can be applied with acceptable results and that our results should be evaluated with larger sample groups and randomized controlled studies.

## Figures and Tables

**Table 1:** Comparison of preoperative, intraoperative, and postoperative processes in patients operated under PNB or Sa.

	PNB	SA
Preoperative Period		
Admission	Day Surgery	Inpatient Admission
Enema	None	None
Fasting	None	6 h Before Surgery
Intraoperative Period		
Position	Lithotomy	Lithotomy
Type of Anesthesia	Isolated Pudendal Block	Standard Spinal Anesthesia
Surgical Procedure	Hemorrhoidectomy: Parks LHP: 980 nm wavelength diode laser, 5 s/shot LAFT: 1470 nm continuous mode Seton: Drainage Seton	Hemorrhoidectomy: Parks LHP: 980 nm wavelength diode laser, 5 s/shot LAFT: 1470 nm continuous mode Seton: Drainage Seton
Surgeon	Same	Same
Postoperative Period		
Postoperative follow up	Recovery Room	Inpatient Ward
Oral Intake	Immediately	Postoperative 4th Hour

**Table 2:** Descriptive analysis results of all patients and subgroups according to anesthesia type.

Diagnosis	PNB (n: 435)	SA (n: 155)	Operation	PNB	SA	Total (n: 590)
Hemorrhoidal Disease (%)	175 (72.6)	66 (27.4)	Hemorrhoidectomy (%)	25 (50)	25 (50)	50
			LHP (%)	133 (78.7)	36 (21.3)	169
			Hemorrhoidopexy (%)	5 (62.5)	3 (37.5)	8
			Thrombectomy (%)	12 (85.7)	2 (14.3)	14
Anal fistula (%)	197 (77)	59 (23)	Fistulotomy (%)	115 (78.2)	32 (21.8)	147
			Seton (%)	76 (75.2)	25 (24.8)	101
			LAFT (%)	6 (75)	2 (25)	8
Anal fissure (%)	28 (66.7)	14 (33.3)	LIS (%)	28 (66.7)	14 (33.3)	42
Anal wart (%)	5 (55.6)	4 (44.4)	Excision cauterization (%)	5 (55.6)	4 (44.4)	9
Anal abscess (%)	30 (71.4)	12 (28.6)	Abscess drainage (%)	30 (71.4)	12 (28.6)	42
Follow-up period/month (iqr)				16 (10–23)	14 (8–25)	16 (9–23)

PNB: pudendal nerve block, SA: spinal anesthesia, LIS: lateral internal sphincterotomy, LAFT: laser ablation of fistula tract, LHP: laser hemorrhoidoplasty, n: number of patients.

**Table 3:** Comparative analysis of VAS scores within and between groups according to disease diagnosis and surgical procedures.

		PNB (iqr)	Intragroup p^f^	SA (iqr)	Intragroup p^f^	p^†^
HD	Hemorrhoidectomy	V^0^ = 4 (2–5)	V^0^-V^1^ > 0.999	V^0^ = 4 (3–6)	V^0^-V^1^ = 0.472	0.317
	n^pnb^: 25, n^sa^: 25	V^1^ = 3 (2–5)	V^0^-V^2^ < 0.001	V^1^ = 5 (4–7)	V^0^-V^2^ < 0.001	<0.001
		V^2^ = 1 (1–2)	V^1^-V^2^ = 0.003	V^2^ = 2 (1–3)	V^1^-V^2^ < 0.001	0.023
	LHP	V^0^ = 5 (6–2.5)	V^0^-V^1^ < 0.001	V^0^ = 6 (3–7)	V^0^-V^1^ > 0.999	0.396
	n^pnb^: 133, n^sa^: 36	V^1^ = 3 (1–4)	V^0^-V^2^ < 0.001	V^1^ = 5 (4–6)	V^0^-V^2^ = 0.033	0.002
		V^2^ = 2 (1–3)	V^1^-V^2^ = 0.023	V^2^ = 1 (0–1)	V^1^-V^2^ = 0.069	0.010
	Hemorrhoidopexy	V^0^ = 4 (3–7)	p^f^ = 0.50	V^0^ = 3 (3–4)	p^f^ = 0.086	>0.999
	n^pnb^: 5, n^sa^: 3	V^1^ = 2 (1–3)		V^1^ = 3 (3–4)		0.071
		V^2^ = 3 (1–3)		V^2^ = 1 (1–2)		0.786
	Thrombectomy	V^0^ = 3.5 (2–5)	p^f^ = 0.192	V^0^ = 6 (5–7.5)	p^f^ = 0.135	0.198
	n^pnb^: 12, n^sa^: 2	V^1^ = 3 (2–4)		V^1^ = 4 (4–5)		0.132
		V^2^ = 2 (1.25–3)		V^2^ = 1 (1–1.5)		0.440
FIA	Fistulotomy	V^0^ = 5 (1–6)	V^0^-V^1^ = 0.005	V^0^ = 5 (1.5–6)	V^0^-V^1^ < 0.001	0.531
	n^pnb^: 115, n^sa^: 32	V^1^ = 6 (5–7)	V^0^-V^2^ < 0.001	V^1^ = 7 (6.25–9)	V^0^-V^2^ = 0.031	<0.001
		V^2^ = 1 (1–2)	V^1^-V^2^ < 0.001	V^2^ = 2 (1–2)	V^1^-V^2^ < 0.001	0.090
	Seton	V^0^ = 5 (1–6)	V^0^-V^1^ = 0.056	V^0^ = 5 (1–6)	V^0^-V^1^ > 0.999	0.753
	n^pnb^: 76, n^sa^: 25	V^1^ = 2 (2–3)	V^0^-V^2^ < 0.001	V^1^ = 4 (4–5)	V^0^-V^2^ < 0.001	<0.001
		V^2^ = 1 (1–2.75)	V^1^-V^2^ = 0.002	V^2^ = 2 (1–3)	V^1^-V^2^ = 0.001	0.067
	LAFT	V^0^ = 3 (0.75–5.75)	p^f^ = 0.405	V^0^ = 2 (2–5)	p^f^ = 0.223	0.429
	n^pnb^: 6, n^sa^: 2	V^1^ = 2 (1–3.75)		V^1^ = 4 (4–4)		0.286
		V^2^ = 2 (0.75–2.5)		V^2^ = 1 (1–1.5)		0.857
AF	LIS	V^0^ = 4 (4–7)	V^0^-V^1^ > 0.999	V^0^ = 4 (3.75–7)	V^0^-V^1^ > 0.999	0.955
	n^pnb^: 28, n^sa^: 14	V^1^ = 4.5 (4–6.75)	V^0^-V^2^ < 0.001	V^1^ = 6 (4.75–8.25)	V^0^-V^2^ = 0.014	0.045
		V^2^ = 1 (1–2)	V^1^-V^2^ < 0.001	V^2^ = 1 (1–2)	V^1^-V^2^ = 0.001	0.966
AW	Excision cauterization	V^0^ = 5 (2.5–7.5)	V^0^-V^1^ = 0.618	V^0^ = 3 (1.25–4.75)	V^0^-V^1^ = 0.102	0.315
	n^pnb^: 5 n^sa^: 4	V^1^ = 7 (5.5–7.5)	V^0^-V^2^ = 0.618	V^1^ = 8.5 (7.25–9)	V^0^-V^2^ > 0.999	0.059
		V^2^ = 3 (1–4.5)	V^1^-V^2^ = 0.034	V^2^ = 3 (1.25–4)	V^1^-V^2^ = 0.102	>0.999
PA	Abscess drainage	V^0^ = 7 (6–8)	V^0^-V^1^ = 0.002	V^0^ = 6 (5.25–8)	V^0^-V^1^ > 0.999	0.314
	n^pnb^: 30, n^sa^: 12	V^1^ = 4 (3–6)	V^0^-V^2^ < 0.001	V^1^ = 6 (5.25–7.75)	V^0^-V^2^ = 0.002	<0.001
		V^2^ = 1.5 (1–2.25)	V^1^-V^2^ = 0.005	V^2^ = 2 (2–3)	V^1^-V^2^ < 0.001	0.129

HD: hemorrhoidal disease, FIA: anal fistula, AF: anal fissure, AW: anal wart, PA: perianal abscess LAFT: laser ablation of fistula tract, LHP: laser Hemorrhoidoplasty, n^pnb^: number of patients operated with pudendal nerve block, n^sa^: number of patients operated under spinal anesthesia, iqr: interquartile range, V^0^: preoperative VAS, V^1^: postoperative 1st-week VAS, V^2^: postoperative 1st-month VAS, iqr: interquartile range, p^f^: Friedman test, p^†^: Mann–Whitney U test.

**Table 4:** Comparative analysis of WHODAS 2.0 total scores within and between groups according to disease diagnosis and surgical procedures.

		PNB (iqr)		Intragroup p^§^	SA (iqr)		Intragroup p^§^	p^†^
HH	Hemorrhoidectomy	D^0^	10.3 (5.4–21.8)	**<0.001**	D^0^	12.6 (5.4–28.8)	**0.009**	0.415
	n^pnb^: 25, n^sa^: 25	D^1^	5.1 (2.6–14.6)		D^1^	13 (7.4–16.8)		**0.009**
	LHP	D^0^	11 (5–27.9)	**<0.001**	D^0^	6.9 (2.9–31.3)	0.413	0.403
	n^pnb^: 133, n^sa^: 36	D^1^	6 (2.4–14.4)		D^1^	10.2 (9.3–22.7)		0.093
	Hemorrhoidopexy	D^0^	11 (4.9–30.5)	**0.043**	D^0^	5.8 (4.1–20)	0.109	0.571
	n^pnb^: 5, n^sa^: 3	D^1^	9.8 (3.1–18.8)		D^1^	8.1 (6.3–21.9)		0.786
	Thrombectomy	D^0^	14.6 (3.7–39.7)	**0.004**	D^0^	21.3–33.4	0.180	0.440
	n^pnb^: 12, n^sa^: 2	D^1^	7.5 (1.4–17.3)		D^1^	14.6–16.8		0.352
FIA	Fistulotomy	D^0^	13.5 (1.9–37.2)	**<0.001**	D^0^	15.6 (3.6–38.5)	**0.018**	0.400
	n^pnb^: 115, n^sa^: 32	D^1^	9.3 (1.9–19.9)		D^1^	13.8 (4.2–23.6)		**0.047**
	Seton	D^0^	11.1 (4.3–25.6)	**<0.001**	D^0^	15.6 (3.3–43.8)	**0.045**	0.549
	n^pnb^: 76, n^sa^: 25	D^1^	5.7 (2.5–18.8)		D^1^	10.6 (5.5–27.2)		**0.029**
	LAFT	D^0^	6.6 (5.1–26.2)	**0.043**	D^0^	5.8–32.8	0.655	>0.999
	n^pnb^: 6, n^sa^: 2	D^1^	3.1 (2.7–9.1)		D^1^	6.9–18.4		0.143
AF	LIS	D^0^	20.8 (20.8–32.8)	**<0.001**	D^0^	21.1 (20.8–32.9)	**0.048**	0.589
	n^pnb^: 28, n^sa^: 14	D^1^	18.5 (14.7–21.8)		D^1^	23.3 (19.7–25.7)		**0.012**
AS	Excision cauterization	D^0^	20.8 (10.4–30.7)	0.080	D^0^	19.9 (17.9–22.9)	0.465	0.905
	n^pnb^: 5 n^sa^: 4	D^1^	17.7 (5.5–24.5)		D^1^	16.3 (8.8–23.4)		0.730

FIA: anal fistula, AF: anal fissure, LAFT: laser ablation of fistula tract, LHP: laser hemorrhoidoplasty, n^pnb^: number of patients operated with pudendal nerve block, n^sa^: number of patients operated under spinal anesthesia, iqr: interquartile range, D^0^: preoperative WHODAS 2.0 score, D^1^: postoperative 1st-month WHODAS 2.0 score, p^§^: Wilcoxon test *p* value, p^†^: Mann–Whitney u test *p* value.

**Table 5:** Comparative analysis of postoperative complications.

	Bleeding			Urinary Retention			SSI			FI (Temporary or Permanent)		
	PNB	SA	p	PNB	SA	p	PNB	SA	p	PNB	SA	p
Hemorrhoidectomy (%)	3 (12)	4 (16)	>0.999	0 (0)	2 (8)	0.490	0 (0)	0 (0)	-	0 (0)	0 (0)	-
n^pnb^: 25, n^sa^: 25												
LHP (%)	2 (1.5)	1 (2.8)	0.515	0 (0)	0 (0)	-	0 (0)	0 (0)	-	0 (0)	0 (0)	-
n^pnb^: 133, n^sa^: 36												
Hemorrhoidopexy (%)	0 (0)	0 (0)	-	0 (0)	2 (66.7)	0.107	0 (0)	0 (0)	-	0 (0)	0 (0)	-
n^pnb^: 5, n^sa^: 3												
Thrombectomy (%)	0 (0)	0 (0)	-	0 (0)	0 (0)	-	0 (0)	0 (0)	-	0 (0)	0 (0)	-
n^pnb^: 12, n^sa^: 2												
Fistulotomy (%)	5 (4.3)	2 (6.3)	0.646	1 (0.9)	3 (9.4)	**0.032**	2 (1.7)	2 (6.3)	0.207	4 (3.5)	2 (6.3)	0.611
n^pnb^: 115, n^sa^: 32												
Seton (%)	1 (1.3)	1 (4)	0.436	0 (0)	3 (12)	**0.014**	2 (2.6)	2 (8)	0.255	0 (0)	0 (0)	-
n^pnb^: 76, n^sa^: 25												
LAFT (%)	0 (0)	0 (0)	-	0 (0)	0 (0)	-	0 (0)	0 (0)	-	0 (0)	0 (0)	-
n^pnb^: 6, n^sa^: 2												
LIS (%)	2 (7.1)	1 (7.1)	>0.999	0 (0)	2 (14.3)	0.106	1 (3.6)	1 (7.1)	>0.999	0 (0)	1 (7.1)	0.333
n^pnb^: 28, n^sa^: 14												
Excision cauterization (%)	0 (0)	1 (25)	0.444	0 (0)	0 (0)	-	1 (20)	1 (25)	>0.999	0 (0)	0 (0)	-
n^pnb^: 5 n^sa^: 4												
abscess drainage (%)	1 (3.3)	1 (8.3)	0.495	1 (3.3)	2 (16.7)	0.192	1 (3.3)	1 (8.3)	0.495	0 (0)	0 (0)	-
Total (%)	14 (3.2)	11 (7.1)	0.050	2 (0.5)	14 (9)	**<0.001**	7 (1.6)	7 (4.5)	0.060	4 (0.9)	3 (1.9)	0.387

LAFT: laser ablation of fistula tract, LHP: laser hemorrhoidoplasty, LIS: lateral internal sphincterotomy, n^pnb^: number of patients operated with pudendal nerve block, n^sa^: number of patients operated under spinal anesthesia, SSI: Surgical Site infection, FI: fecal incontinence

**Table 6:** Comparative analysis of operation time and hospital stay.

		Pudental (±SD)	Spinal (±SD)	p §
Hemorrhoidectomy	OD/min	27.8 (5.2)	40.64 (6.1)	**<0.001**
	HD/h	1.7 (0.8)	29.4 (8.9)	**<0.001**
LHP	OD/min	22.9 (4.9)	42.3 (8)	**<0.001**
	HD/h	1.4 (0.6)	28.6 (9.3)	**<0.001**
LIS	OD/min	15.6 (1.8)	30.7 (1.9)	**<0.001**
	HD/h	1.6 (0.8)	27.2 (6.5)	**<0.001**
Seton	OD/min	24.6 (5.9)	41.2 (7.4)	**<0.001**
	HD/h	1.6 (0.8)	27.7 (5.2)	**<0.001**
Fistulotomy	OD/min	19.8 (5.31)	36.3 (4.6)	**<0.001**
	HD/h	1.6 (0.8)	27.6 (5.1)	**<0.001**

LHP: laser hemorrhoidoplasty, LIS: lateral internal sphincterotomy, OD: operation duration, HD: hospitalization duration, min: minute, h: hour, SD: standard deviation, §: independent sample *t* test.

## Data Availability

No data are available due to privacy or ethical restrictions.

## Supplementary Materials

The following supporting information can be downloaded at https://www.mdpi.com/article/10.3390/medicina60101651/s1, Supplementary file S1: STROBE Statement—checklist of items that should be included in reports of observational studies.
